# Genetic Testing Enables the Diagnosis of Familial Hypercholesterolemia Underdiagnosed by Clinical Criteria: Analysis of Japanese Early-Onset Coronary Artery Disease Patients

**DOI:** 10.1155/2023/2236422

**Published:** 2023-04-28

**Authors:** Hiroshi Miyama, Yoshinori Katsumata, Mizuki Momoi, Genki Ichihara, Taishi Fujisawa, Jin Endo, Takashi Kawakami, Masaharu Kataoka, Shinsuke Yuasa, Motoaki Sano, Kazuki Sato, Keiichi Fukuda

**Affiliations:** ^1^Department of Cardiology, Keio University School of Medicine, Tokyo, Japan; ^2^Institute for Integrated Sports Medicine, Keio University School of Medicine, Tokyo, Japan; ^3^Second Department of Internal Medicine, University of Occupational and Environmental Health, Kitakyushu, Japan

## Abstract

Definitive diagnosis of familial hypercholesterolemia (FH) is paramount for the risk management of patients and their relatives. The present study aimed to investigate the frequency of gene variants contributing to low-density lipoprotein cholesterol (LDL-C) metabolism and their clinical relevance in patients with early-onset coronary artery disease (EOCAD). Among 63 consecutive patients with EOCAD (men <55 years or women <65 years) who underwent percutaneous coronary intervention (PCI) from 2013 to 2019 at Keio University Hospital, 52 consented to participate in this retrospective study. Targeted sequencing of *LDLR*, *PCSK9*, *APOB*, and *LDLRAP1* was performed. Of the 52 patients enrolled (42 men; mean age: 50 ± 6 years), one (*LDLR*, c.1221_1222delCGinsT) harbored a pathogenic mutation, and one (*APOB*, c.10591A>G) harbored variants of uncertain significance. Both the patients harboring the variants were male, showing no history of diabetes mellitus or chronic kidney disease, no family history of EOCAD, and no physical findings of FH (i.e., tendon xanthomas or Achilles tendon thickening). Patients harboring the *LDLR* variant had three-vessel disease, were on a statin prescription at baseline, and had stable LDL-C levels; however, the case showed a poor response to the intensification of medication after PCI. Approximately 3.8% of patients with EOCAD harbored variants of gene related to LDL-C metabolism; there were no notable indicators in the patients' background or clinical course to diagnose FH. Given the difficulty in diagnosing FH based on clinical manifestations and family history, genetic testing could enable the identification of hidden risk factors and provide early warnings to their relatives.

## 1. Introduction

Familial hypercholesterolemia (FH) is a prevalent autosomal dominant disorder that is mainly caused by a variant in the *LDLR*, *PCSK9*, or *APOB* gene, resulting in abnormal low-density lipoprotein cholesterol (LDL-C) metabolism. It is known as an important trigger of early-onset coronary artery disease (EOCAD) [1–5]. Early intervention and control of LDL-C levels in patients with FH can reduce cardiovascular events [6]. Therefore, determination of the genetic background of patients and strict management of the family profile are of vital importance.

Although evaluation of clinical diagnostic criteria is the cornerstone for diagnosing FH in daily clinical practice, patients with genetically diagnosed FH who do not meet the criteria are still at high risk of coronary artery disease (CAD) [7]. A survey revealed that less than half of patients with CAD who had definite or probable clinical FH showed pathogenic variants in FH-related genes [8, 9]. This discrepancy in clinical and genetic diagnoses can be partly explained by the fact that CAD triggers are largely environmental in origin, besides those of genetic origin [10–13]. Considering the necessity of risk stratification for family members, genetic diagnosis of FH in patients with EOCAD would be of utmost importance. In an era in which genetic tests can be performed easily and rapidly, reliance on clinical criteria is insufficient. However, very little is known about the exact prevalence and characteristics of genetically diagnosed FH in the general population with EOCAD.

The present study aimed to conduct a genetic analysis related to LDL-C metabolism and investigate the clinical characteristics of patients with EOCAD treated at a single institution in Tokyo.

## 2. Materials and Methods

### 2.1. Study Cohort

**Figure 1:**
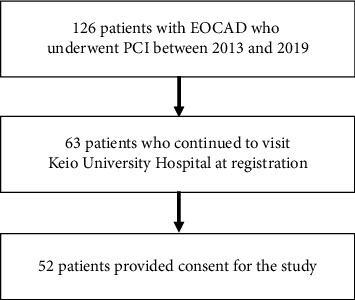
Flow diagram showing patient enrolment. CAD, coronary artery disease; PCI, percutaneous coronary intervention.

A total of 126 patients with EOCAD (men <55 years and women <65 years of age at the time of percutaneous coronary intervention (PCI)) underwent index PCI for CAD (acute coronary syndrome, exertional angina, and asymptomatic myocardial ischemia) from 2013 to 2019 at Keio University Hospital in Tokyo, Japan. Among the patients, 63 continued to visit the hospital at the time of study registration, and 52 out of the 63 provided informed consent for study enrolment. In this study, we used oral epithelial cells for genomic analysis targeting the following four genes related to LDL-C metabolism: *LDLR*, *PCSK9*, *APOB*, and *LDLRAP1* (Figure 1).

This study was conducted in accordance with the tenets of the Declaration of Helsinki, and approval was obtained from the institutional review board/ethics committee. The research was registered in the University Hospital Medical Information Network (registration number: UMIN000039852). All participants provided written informed consent.

### 2.2. Sequencing and Variant Selection

Sequencing was performed using an Illumina MiSeq sequencer (Illumina Inc., San Diego, CA, USA). Alignment against the reference sequence was performed using BWA-MEM [14], and variant calling was performed using VarScan. Variants with a minor allele frequency (MAF) below 1% were considered within a potential pathogenic range, and a quality score (*Q*) of 30 or higher was adopted. Synonymous variants were excluded if they were demonstrated to be nonpathogenic or benign. Variants were also predicted *in silico* using PolyPhen-2 and SIFT scores, as well as the combined annotation-dependent depletion (CADD) scoring model with a cut-off value of 15 as a computational prediction [15, 16]. Finally, we defined a causative variant for FH based on whether it fulfilled any of the following criteria: (a) rare (MAF below 1% in the East Asian population) protein-truncating variants (premature stop, frame-shifting insertions or deletions, or canonical splice-sites) at the *LDLR* gene; (b) rare damaging missense variants at the *LDLR*, *PCSK9*, or *APOB* gene (i.e., those predicted to be damaging by all three *in-silico* programs, namely, PolyPhen-2, SIFT score, and CADD scoring model); and (c) ClinVar-registered pathogenic or likely pathogenic variants, related to FH, in the *LDLR*, *PCSK9*, or *APOB* gene. In addition, we evaluated whether the variants were classified as pathogenic, with supporting evidence based on the American College of Medical Genetics (ACMG) criteria [17].

### 2.3. Clinical Evaluations

Patients with variants were compared with respect to their clinical background, including past medical history and family history of EOCAD (men <55 years and women <65 years of age at the time of CAD diagnosis). Hypertension was defined as systolic blood pressure ≥140 mmHg, diastolic blood pressure ≥90 mmHg, or prior use of antihypertensive medication. Diabetes mellitus was defined according to the diagnostic criteria described by the Japan Diabetes Society. Physical examination was used to confirm Achilles tendon thickening, tendon xanthoma, or corneal arcus. Data were expressed as median (interquartile range (IQR)) or frequency (percentage), depending on whether the variable was considered continuous or categorical, respectively.

## 3. Results

### 3.1. Study Population

The majority (80.8%, *n* = 42) of patients were men, and the median age was 50 years (IQR, 46–53). Ten patients (19.2%) had a family history of EOCAD. The mean baseline LDL-C level was 125 mg/dL (IQR, 114–158), and 13 patients (25.0%) had already been prescribed statins or ezetimibe for hypercholesterolemia at the time of enrolment.

### 3.2. Genetic Characteristics

From 52 genomic DNA samples, we sequenced four genes (namely, *LDLR*, *PCSK9*, *APOB*, and *LDLRAP1*), including exonic and splicing regions. We identified two variants, one of which was predicted to be harmful based on the CADD score. Overall, one patient showed pathogenic variants (PM: *LDLR*, c.1221_1222delCGinsT), and another showed variants of uncertain significance (VUS: *APOB*, c.10591A>G) (Table 1). No variants of *PCSK9* and *LDLRAP1* were detected. In our EOCAD cohort, none of the variants showed duplications or were observed together in a single case.

### 3.3. Association between Genotype and Phenotype

Table 2 shows the comparison of baseline characteristics between patients harboring the variants and those from the total cohort. The proportion of men in the overall cohort was 80.8% (42 cases), and 10 cases (19.2%) had a family history of EOCAD. Both the patients harboring the variants were male, and neither of them had a family history of EOCAD. Although both patients were smokers, there were no environmental causes, such as diabetes mellitus or chronic kidney disease in either of the patients. Patients harboring the variants did not show FH-specific physical manifestations, such as tendon xanthomas or Achilles tendon thickening. The patient harboring the *LDLR* variant was treated with a statin at baseline, and his LDL-C level was relatively stable (119 mg/dL). However, he showed three-vessel disease and the intensified medication regimen after PCI was not sufficient to lower the LDL-C level, suggesting refractoriness (Table 2).

## 4. Discussion

In this retrospective study, approximately 3.8% of patients with EOCAD had gene variants related to LDL-C metabolism. For the patients harboring the variants, only a few environmental factors contributed to the disease. In addition, there was no obvious clinical manifestation or a family history of EOCAD, based on the clinical diagnostic criteria. Thus, we found that genetic testing can not only identify patients with FH and reinforce individual risk management but also stratify the hidden risk of cardiovascular events and alert relatives to a potential need for intervention.

In daily clinical practice, FH is mainly diagnosed based on clinical diagnostic criteria (e.g., Dutch Lipid Clinic Network criteria (DLCNC), Simon Broome criteria, and Japan Atherosclerosis Society criteria). These criteria comprise of a family history of EOCAD, physical findings, and serum LDL-C levels [18–20]. Nanchen et al. have reported that among young patients with acute coronary syndromes in Switzerland, 1.6% had probable or definite FH, according to the clinical diagnostic criteria [21]. Regarding the differences between genetic diagnoses, some reports have shown a high diagnostic accuracy of clinical diagnostic criteria compared to that of genetic diagnosis, whereas others have shown discrepancies between the two [22, 23]. Although there are only a few reports of genetic testing in young CAD patients, the prevalence of FH-associated variants shows a relatively wide range depending on the type of gene targeted, the criteria used to determine the pathogenicity of the variants, and racial differences (2–21%) [4, 5]. It is necessary to develop further evidence, establish methods for identifying the pathogenicity of variants, improve comprehensive genetic analysis (including structural variations), and construct unified diagnostic criteria when making a genetic diagnosis of FH in EOCAD patients. Despite being an inexpensive and efficient tool to diagnose FH, clinical diagnosis alone may cause a certain number of patients with FH to be overlooked [7, 23]. In the present study, the two cases that tested genetically positive were clinically undiagnosed cases, showing relatively low or controlled LDL-C levels and no family history of EOCAD. Such underdiagnoses can lead to the overestimation of cardiovascular prognosis in patients with FH and their family members.

Genetic testing has recently become both inexpensive and rapid, and it is a feasible approach for patients with CAD. Combination of genetic testing with conventional clinical diagnostic criteria can improve the risk stratification of cardiovascular events in patients with hypercholesterolemia, suggesting an additive effect and true clinical importance of aggressive genetic testing [7]. Genetic testing is important not only for the assessment of cardiovascular risks or treatment strategies for patients with FH themselves but also for risk management in family members. The risk of CAD events in patients has been associated with cumulative LDL-C levels (mg/dL × years) [3]. This can be efficiently reduced by early intervention [6, 24, 25]. Therefore, verification of the risk of FH among relatives of patients with EOCAD and, if necessary, diagnosis using genetic testing can be a cost-effective strategy to prevent the disease in the active age group and can improve lifetime cardiovascular prognosis.

Naturally, genetic testing has certain limitations; for example, the number of candidate variants for FH is gradually increasing [26–28]. The current standard for determining pathogenicity is to refer to the ACMG guidelines; variants with established pathogenicity can be considered responsible for FH. However, we often see previously unreported variants, and their clinical relevance is difficult to evaluate. In our study, we found previously unreported variants of *LDLR* (c.1221_1222delCGinsT) and *APOB* (c.10591A>G). The pathogenicity of the variant detected in *APOB* was not definitive, but it was predicted to be damaging based on *in-silico* analysis and was indicated to have a high level of predicted pathogenicity, according to the ACMG guidelines [17]. The variant in *LDLR* was a truncated rare variant, which was also considered to be a highly pathogenic variant. Another factor that can confuse the decision-making process is a structural variant of the contributing variant itself. This would make it difficult to accurately diagnose using a conventional panel analysis, exome analysis, or whole genome analysis. Continued accumulation of functional analysis data and construction of a genetic database focusing on FH, including racial differences, would be helpful in overcoming these challenges.

The current study had several limitations. First, it was conducted retrospectively at a single center. Therefore, unmeasured confounding factors, such as mental disorders, FH-specific physical findings, or economic status, may have been present. Second, the number of patients who dropped out of the cohort was large due to the nature of the institution; patients are often referred to other medical facilities once conditions are deemed chronic. Third, no functional analysis was performed to validate the pathogenicity of FH-linked genetic variants in the present study; this is particularly desirable for the evaluation of *APOB* variants. We did not identify pathogenic variants in individuals with confirmed FH. We were not able to assess all relatives of patients with confirmed or suspected FH, despite our vigorous cascade screening. A lack of data might have led to a biased assessment. Fourth, the LDL-C level before statin administration was not available in the case with the *LDLR* variant. However, clinically implicating FH remained difficult, considering that the LDL-C level was relatively stable after treatment with 5 mg of rosuvastatin at the time of initial presentation. Fifth, the genetic analysis performed in the present study did not include the possible detection of large deletions or duplications in the *LDLR* gene, which may contribute to FH to some degree. Finally, the study was performed exclusively with Japanese patients; the results might not be applicable to other ethnicities.

## 5. Conclusions

Approximately 3.8% of patients with EOCAD had FH-related gene variants that were not diagnosed using clinical diagnostic criteria. Genetic testing can accurately identify patients with FH who present few clinical findings other than CAD and can reinforce risk management; moreover, it can stratify the cardiovascular risk of relatives and suggest intervention to prevent adverse outcomes.

## Figures and Tables

**Table 1:** List of genetic variants.

Gene	Chromosome	Nucleotide change	Amino acid change	Count	Variant type	*In silico*			ACMG criteria	Variant class
						PolyPhen-2	SIFT score	CADD score		
*LDLR*	19	c.1221_1222delCGinsT	p.Glu408ArgFs*∗*5	1	Nonsense	NA	NA	NA	PVS1 + PM2	PM
*APOB*	2	c.10591A>G	p.Lys3531Glu	1	Missense	0.574	0	19.36	PM1 + PM2 + PM6	VUS

CADD score, combined annotation-dependent depletion score; ACMG criteria, American college of medical genetics criteria; PM, pathogenic mutation; VUS, variant of uncertain significance.

**Table 2:** Baseline characteristics.

Variables, *n* (%)	Total population (*n* = 52)	Case 1	Case 2
Responsible gene	NA	*LDLR*	*APOB*
CAD type	NA	EAP	UAP
Age, years (IQR)	49 (46–53)	59	39
Male	42 (80.8)	Male	Male
BMI, (kg/m^2^) (IQR)	25.1 (23.5–27.7)	24.5	21.9
Family history of early-onset CAD	10 (19.2)	No	No
Medical history			
Smoking	33 (63.5)	Yes	Yes
Hypertension	25 (48.1)	No	Yes
Diabetes mellitus	14 (26.9)	No	No
Stroke	3 (5.8)	No	No
Peripheral artery disease	2 (3.8)	No	No
Three-vessel disease	15 (28.8)	Yes	No
Left main trunk lesion	3 (5.8)	No	No
CAD relapse	6 (11.5)	No	No
Laboratory findings at baseline			
LDL-C, (mg/dL) (IQR)	124 (112–163)	119	108
HDL-C, (mg/dL) (IQR)	43 (37–48)	62	37
Triglycerides, (mg/dL) (IQR)	152 (98–225)	98	151
HbA1c, % (IQR)	5.9 (5.4–6.4)	6.0	5.9
Creatinine, (mg/dL) (IQR)	0.80 (0.67–1.01)	1.0	0.85
eGFR, mL/min/1.73 m^2^ (IQR)	77 (60–88)	62	81
Laboratory findings after medications			
LDL-C, (mg/dL) (IQR)	81 (65–96)	121	75
HDL-C, (mg/dL) (IQR)	46 (40–54)	77	47
Triglycerides, (mg/dL) (IQR)	146 (111–178)	49	101
Changes in LDL-C levels, (mg/dL) (IQR)	−50 (−73 to −23)	2	−33
Baseline prescriptions			
Statins	12 (23.1)	Rosuvastatin, 5 mg	No
Ezetimibe	1 (1.9)	No	No
Present prescriptions			
Statins	51 (98.1)	Rosuvastatin, 10 mg	Rosuvastatin, 5 mg
Ezetimibe	18 (34.6)	No	Yes
EPA	2 (3.8)	No	No
PCSK-9 inhibitor	1 (1.9)	No	No

PM, pathogenic mutation; VUS, variant of uncertain significance; EAP, effort angina pectoris; UAP, unstable angina pectoris; BMI, body mass index; CAD, coronary artery disease; eGFR, estimated glomerular filtration rate; LDL-C, low-density lipoprotein cholesterol; HDL-C, high-density lipoprotein cholesterol; HbA1c, hemoglobin A1c; EPA, eicosapentaenoic acid; PCSK-9, proprotein convertase subtilisin/kexin type 9; IQR, interquartile range.

## Data Availability

The data supporting the current study are available from the corresponding author upon request.
